# Mechanisms of Nerve Capping Technique in Prevention of Painful Neuroma Formation

**DOI:** 10.1371/journal.pone.0093973

**Published:** 2014-04-04

**Authors:** Hede Yan, Feng Zhang, Jon Kolkin, Chunyang Wang, Zhen Xia, Cunyi Fan

**Affiliations:** 1 Department of Orthopedics, Shanghai Jiao Tong University Affiliated Sixth People's Hospital, Shanghai, China; 2 Department of Orthopaedics (Division of Plastic and Hand Surgery), The Second Affiliated Hospital of Wenzhou Medical University, Wenzhou, China; 3 Division of Plastic Surgery, University of Mississippi Medical Center, Jackson, Mississippi, United States of America; 4 Department of Plastic and Hand Surgery, Duke Raleigh Hospital, Raleigh, North Carolina, United States of America; University of Arizona, United States of America

## Abstract

Nerve capping techniques have been introduced as a promising treatment modality for the treatment of painful neuroma with varied outcomes; however, its exact mechanism is still unknown. RhoA is one of the members of the RAS superfamily of GTPases that operate as molecular switches and plays an important role in peripheral nerve regeneration. Our aim was to investigate the structural and morphologic mechanisms by which the nerve capping technique prevents the formation of painful neuromas after neuroectomy. We also hoped to provide a theoretical basis for this treatment approach. An aligned nanofiber conduit was used for the capping procedure and the sciatic nerve of Sprague-Dawley rats was selected as the animal model. Behavioral analysis, extent of neuroma formation, histological assessment, expressions of pain markers of substance P and c-fos, molecular biological changes as well as ultrastructural features were investigated and compared with the findings in a no-capping control group. The formation of traumatic neuromas was significantly inhibited in the capping group with relatively “normal” structural and morphological features and no occurrence of autotomy and significantly lower expression of pain markers compared to the no-capping group. The gene expression of RhoA was consistently in a higher level in the capping group within 8 weeks after surgery. This study shows that capping technique will alter the regeneration state of transected nerves and reduce painful neuroma formation, indicating a promising approach for the treatment of painful neuroma. The initiation of the “regenerative brake” induced by structural as well as morphological improvements in the severed nerve is theorized to be most likely a key mechanism for the capping technique in the prevention of painful neuroma formation.

## Introduction

Traumatic neuroma formation is a major cause of neuropathic pain, which is still a challenging problem faced by surgeons [1], [2]. Although the exact mechanism of neuroma-associated pain is not yet fully understood, prevention of neuroma formation is paramount for the prevention of neuropathic pain [3]. Various techniques have been described to minimize neuroma formation with variable outcomes [4]–[7]. To date, the most promising and practical method of neuroma treatment has been surgical removal and transplantation of the nerve stump into a vein, the so-called nerve capping technique [4], [8]. However, its usage is limited by the size of available veins [9]. Therefore, a variety of synthetic materials have been developed for this purpose [10]–[15]. It is speculated that the nerve capping technique allows for epineurial healing over the severed fascicles within the chamber, lessening improperly and irregularly regenerating nerve fibers, thus preventing the formation of traumatic neuromas. However, results using different capping materials have been inconsistent [2], [13] and little is known regarding the exact mechanism of this technique.

The maturity of regenerated nerve fibers in the neuroma plays an important role in the pathology of traumatic neuroma [16]–[18]. MAG, MBP and PMP22 are myelin-specific genes and are significantly upregulated during Schwann cell myelination. NCAM-1, on the other hand, is associated with immature Schwann cells and is often down-regulated during myelination [19], [20]. In vitro study has shown that aligned electrospun fibers significantly upregulated the expression of MAG, MBP and PMP22, and downregulated the expression of NCAM-1, suggesting the propensity of aligned fibers in promoting Schwann cell maturation [21]. However, no in vivo investigations have been reported regarding the impact of aligned biomaterials on the myelination status after nerve injuries.

RhoA is one of the members of the RAS superfamily of GTPases that operates as a molecular switch and contributes to cell polarity and asymmetry [22]. In the central nervous system, RhoA GTPase signaling through Rho kinase (ROK) promotes growth cone collapse and inhibits its regrowth [23]. In the peripheral nervous system, RhoA GTPase was apparently expressed and further up-regulated in response to injury within peripheral neurons, presenting greater axon outgrowth when RhoA-ROK signaling is inhibited [24]. Therefore, the knowledge of the gene expression changes of RhoA may provide new insights into the prevention and treatment of traumatic neuroma.

In our previous study, we designed an aligned absorbable nanofiber conduit, which was fabricated using aligned silk fibroin (SF) blended with poly(L-lactic acid-co-ε-caprolactone) (P(LLA-CL)) nanofibrous scaffolds and achieved satisfactory results in peripheral nerve regeneration in a rat model [25]. In this study, we hypothesize that application of this aligned nanofiber nerve conduit (SF/P(LLA-CL)) will enhance linear nerve outgrowth, accelerate the maturity of regenerated Schwann cells and upregulate the gene expression of RhoA, thereby inducing a nerve growth “ stop signal” to inhibit the process of posttraumatic neuroma formation, and therefore, prevent neuropathic pain after neurectomy.

## Materials and Methods

### Animal models and grouping

Four-eight male Sprague-Dawley rats weighing 250–300 g were used in this study and randomly divided into a no-capping group (n = 24) and a capping group using aligned SF/P(LLA-CL) nanofiber conduits (n = 24). All experiments were approved by the Institutional Animal Care and Use Committee, Shanghai Jiaotong University School of Medicine. Animals were treated according to the National Research Council's guidelines for the care and use of laboratory animals and had free access to rat chow and water.

### Surgical procedure

**Figure 1:**
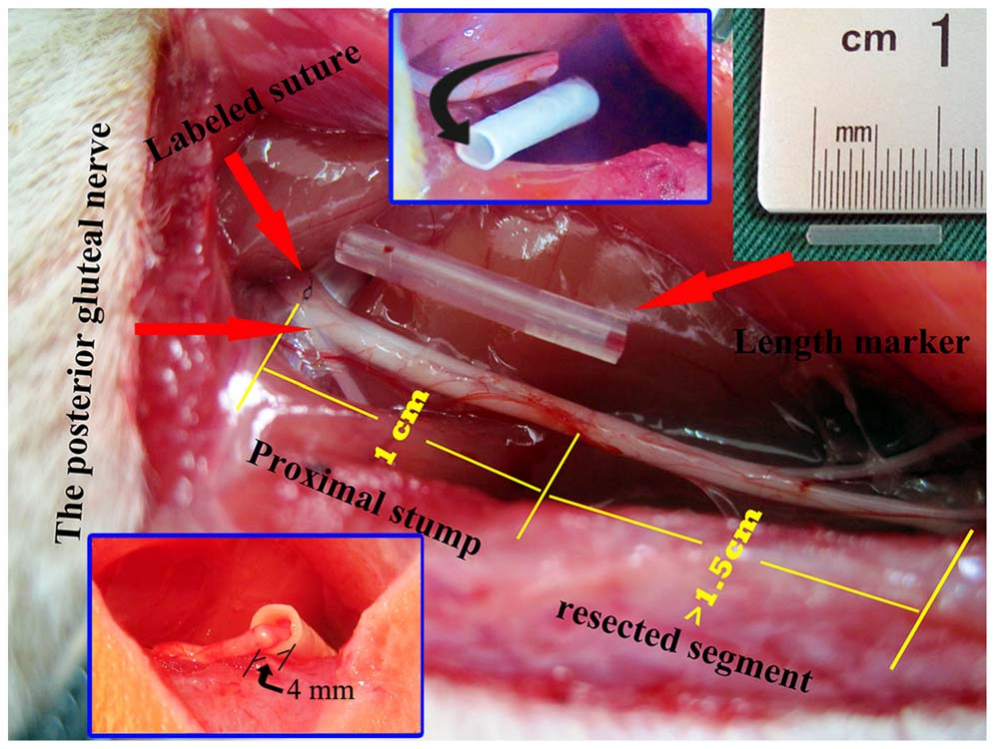
Demonstration of surgical procedures. The red arrows on the left show the site where the labeled suture was placed for quantitative analysis (top) and the origin of the posterior gluteal nerve (bottom); the arrow on the right indicates the length maker (the silicone tube in the middle) for accurate nerve cut. The numbers of 1 and 1.5 in yellow and the inset pictures (above, middle and below, left) show the details of preparation of proximal nerve stump.

Each rat was anesthetized with an intraperitoneal injection of sodium pentobarbital (50 mg/kg), and the right sciatic nerve was exposed under aseptic conditions. In order to achieve a quantitative analysis of neuroma growth, the site giving off the branch of the posterior gluteal nerve (PGN) around the level of the sciatic notch was identified and labeled with a 7–0 suture under microscopy. The sciatic nerve was then sharply transected 1 cm distal to the labeled site with the assistance of a 1 cm-long marker and separated from the surrounding tissue up to the labeled site. In all cases, a gap of at least 1.5 cm was maintained distal to the transection site in order to avoid spontaneous nerve regeneration. In the capping group, the proximal nerve stump was inserted 4 mm into the 1.5 cm-long SF/P(LLA-CL) nanofiber conduit (internal diameter, 1.5 mm), and was sutured to the conduit with a single epineurial 11–0 monofilament nylon suture. In the no-capping group, the proximal nerve stump was not covered by a conduit but also a stitch was performed at the same level as in the capping group. In both groups, muscle wound beds and skin incisions were closed with 4–0 sutures and Ibuprofen analgesia was administered daily for 1 week postoperatively. (Fig. 1)

### Animal sacrificing time and specimen preparation

Half of the rats (n = 12) in each group were randomly divided into 2-week and 8-week subgroups and sacrificed at 2 and 8 weeks after surgery, respectively. The rats in the 8-week subgroup were used for behavioral analysis. The proximal nerve stump (PNS) was cut from the level of the marked site (the origin of PGN) together with a 1 cm-long segment of normal nerve from the corresponding part of the contralateral side. Of the 12 specimens harvested at 2 weeks in each group, 6 ones were randomly selected for histological studies and the other 6 ones, divided into two parts in the middle longitudinally, were used for quantitative RT-PCR study for the gene expression of MAG, MBP, PMP22 and NCAM-1 and Western-blot analysis of the level of substance P, NGF, TGF-β1, collagen I and III, respectively. At 8 weeks, as prepared at 2 weeks, 6 specimens were used for histological studies; of the other 6 ones, about 1 mm×1 mm segment was first harvested in the center of the neuroma under microscopy visualization for transmission electron microscope study before dividing them into two parts for PCR and western blot analysis. In addition, the ipsilateral and contralateral L4 dorsal root ganglions were harvested for RT-PCR analysis of the gene expression of RhoA and the dorsal horn of the fourth lumbar spinal cord (positioned by the L 4 nerve root) was also harvested for western blot analysis of the expression of c-fos, which was used as a pain marker. All of the specimens for RT-PCR and Western-blot analyses were stored at −80^∘^C.

### Behavioral analysis

The rats in each group (n = 12) were monitored by dual, blinded observers for evidence of neuropathic pain for 8 weeks (autotomy scores recorded 3 times/wk). A modified Wall scale [[26]] was adopted to assign points according to the severity of autotomy. Briefly, 1 point was assigned for 2 or more toenails (maximum, 1 point per limb) and 1 point was assigned for each half-digit (distal and proximal phalanges) for a possible maximum of 10 points per limb.

### Evaluation of neuroma growth

In order to quantitatively analyze the growth differences between groups, we labeled the nerve stump 1 cm proximal to the transection site with a 7–0 suture, where the specimen would be harvested at the end of the experiment. A 1 cm-long corresponding nerve segment in the contralateral site would be obtained for comparison. Then, the neuroma growth was evaluated by a weight ratio (WR) using the formula as: [the weight of neuroma (NW, after removal of the conduit) – the weight of the excised normal nerve segment (NNW)]/NNW.

### Histologic analysis

After the excised proximal stumps were weighed, the specimens were then processed by standard paraffin-embedding methods. Sections were cut at 4 um. In order to standardize the site for immunohistochemical staining, the sections were randomly selected at a distance ranging from 400 to 600 um from the distal ending of the specimens. At least 30 sections were obtained from each sample after the exclusion of small sections or poorly cut sections. Ten sections were randomly selected and half of the sections were used for Trichrome Masson's staining or labeled with antineurofilament (NF200) antibodies, respectively. Subsequent incubation with biotinylated secondary antibodies, horseradish-peroxidase–conjugated avidin-biotin mixture, and chromagenic peroxidase substrate provided visualization of primary antibodies. Chaotic arrangement of minifascicles labeled by NF 200 was assessed semiquantitatively using a method similar to that proposed by Koch et al. [27] In brief, the specimens were graded from few (+) to many (+++) axons (x 400). All immunohistochemical supplies, including primary antibodies, were purchased from Sigma (Sigma, USA).

### Real-Time Quantitative PCR

Total RNA was extracted from the stored specimens using an RNeasy Mini Kit according to the manufacturer's protocol. cDNA was synthesized using a cDNA Reverse Transcription Kit and real-time PCR was performed using SYBR Green Premix Ex Taq (TaKaRa) on an Applied Biosystems Stepone real-time PCR System. All reactions were run in triplicate. The relative expression of each mRNA was calculated using the comparative 2−ΔΔCt method and was normalized againstβ-actin. All data were expressed as mean ± SD. All primers were listed in Table 1.

**Table 1:** List of primers used for RT-PCR studies.

Gene	Forward primer	Reverse primer	Size
MAG	5- agacctgggcctacgaaact -3	5- caccatgcagctgacctcta -3	205 bp
MBP	5- aaccactctggaaagcgaga -3	5- ttctttgggtctgctgtgtg -3	218 bp
PMP22	5- tcctcatctgtgagcgaatg -3	5- acagaccagcaaggatttgg -3	163 bp
NCAM-1	5- tgtgaggtctttgcctaccc -3	5- caccgctgtgcagttgtagt -3	171 bp
RhoA	5- tggtgatggagcttgtggta -3	5- agaggcctcagacggtcata -3	183 bp
β-actin	5- cctagacttcgagcaagaga-3	5- agaggtctttacggatgtca-3	221 bp

### Western Blot Analysis

The specimens were lysed with lysis buffer (100 mmol/L dithiothreitol, 50 mmol/L Tris-HCl, pH 6.8, 2% SDS, and 10% glycerol) containing protease inhibitors. The BCA method was used to determine the total protein concentration. Lysates with equal amounts of protein were resolved on SDS-PAGE, and then transferred to a PVDF membrane. The membrane was blocked with 5% non-fat dry milk in TBST buffer (100 mmol/L NaCl, 50 mmol/L Tris-HCl, pH 7.4 and 0.1% Tween-20) at 4°C overnight. The membranes were washed with TBST buffer and then incubated with monoclonal antibodies against rabbit anti-substance P(dilution: 1∶500, GeneTex, Inc., USA), mouse anti-c-fos (dilution: 1∶200, Santa Cruz Biotechnology, USA), mouse anti-NGF (dilution: 1∶200, Santa Cruz Biotechnology, USA), rabbit anti- TGF-β1 (dilution: 1∶300, Santa Cruz Biotechnology, USA), mouse anti-collagen I (dilution: 1∶300, Santa Cruz Biotechnology, USA) and III (dilution: 1∶200, Boster, Wuhan, China) at room temperature for 1.5 h. After washing, the samples were incubated for 2 hr at room temperature with HRP-conjugated goat anti-mouse IgG (1∶8 000) or goat anti-rabbit IgG(1∶5000). The membrane was then washed with buffer and the image scanned with a GS800 Densitometer Scanner. Optical density data were analyzed using PD Quest 7.2.0 software. Beta-actin (dilution: 1∶1000, Santa Cruz Biotechnology, USA) was used as a control.

### Transmission electron microscopy (TEM) study

The specimens harvested were immediately fixed in 2.5% glutaraldehyde in 0.1 M phosphate buffer overnight and then postfixed in osmium tetroxide 1%, stained with uranyl acetate, dehydrated in acidified 2,2-dimethoxypropane, and embedded in epoxy resin. Ultra-thin sections (silver in color) were cut in an ultra-microtome (LKB 8800) and mounted on grids and examined in a transmission electron microscope. For quantitative analysis, photographs of each nerve sections were taken from 10 random fields of each section and morphometric indices, including thickness of the myelin sheath, the ratio of ummyelinated and myelinated nerve fibers and percentage of fibroblasts of the total cell population, were evaluated using Image J software. All measurements were performed by an investigator who was unaware of the grouping information of each section.

### Statistical Analysis

Nonparametric method of Mann-Whiteney U test was used for the statistical analysis between groups using SPSS v. 19.0. For the autotomy score, repeated measures ANOVA were used for calculating the overall significance of each curve and for comparison of data obtained at different periods after surgery among the groups of animals. The difference in the number of cases with autotomy between the two groups was compared using the chi square test. Data were expressed as mean ± standard deviations. Statistical significance was considered at the 5% level.

## Results

### Autotomy observation

**Figure 2:**
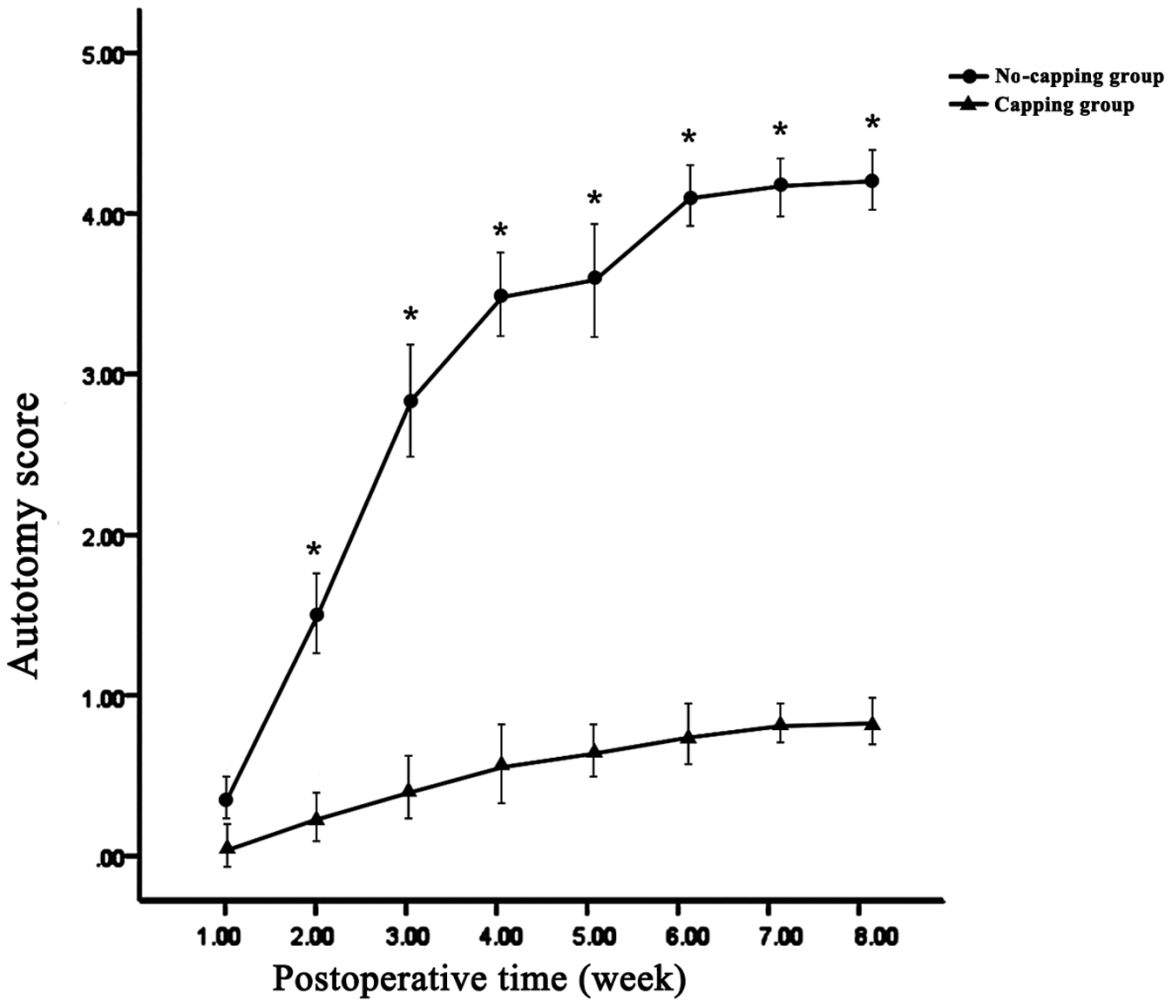
Results of weekly average autotomy scores. Statistically significant differences in the average autotomy score were noted between the two groups at all of the time points (*all p<0.01 vs capping group) except at the end of the first week (p = 0.140).

Autotomy behavior was all noted on the operated side (right foot). Statistically significant differences in the average autotomy score were noted between the two groups at all of the time points (all p<0.01) except at the end of the first week (p = 0.140). (Fig. 2) In addition, the occurrence of autotomy in the capping group was significantly lower than that in the no-capping group: 9 animals in the no-capping group (75%) developed varying degrees of autotomy (autotomy score 3–7, 4.25±2.67); in contrast, only 2 rats in the capping group (16.7%) showed evidence of autotomy. In 81.8% (9/11) of all animals the onset of autotomy occurred between 2 and 4 weeks.

### Neuroma growth

**Figure 3:**
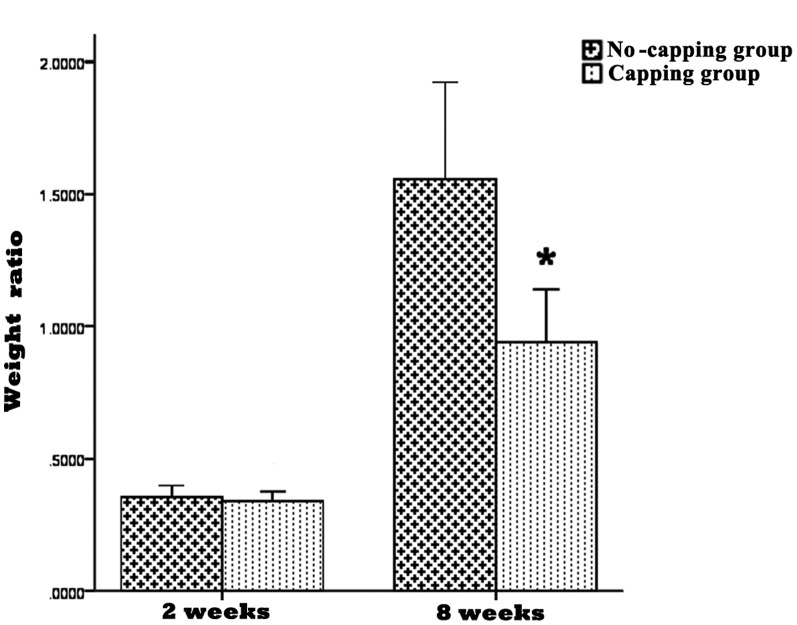
Results of weight ratios (WRs) of neuromas. No significant difference of WR was seen between the two groups at 2 weeks (p = 0.071); however, a significant higher WR was noted in the no-capping group at 8 weeks (*p<0.001 vs capping group).

The WRs of neuromas in the no-capping and capping groups were 0.355±0.022 and 0.339±0.019 at 2 weeks and 1.558±0.183 and 0.941±0.0199 at 8 weeks, respectively. No significant difference of WR was seen between the two groups at 2 weeks (p = 0.071); however, a much higher WR was noted in the no-capping group than in the capping group at 8 weeks (*p<0.001). (Fig. 3)

### General observation and histological findings

At 2 weeks after neuroectomy, a slightly bulb-like neuroma was found in the end of the PNS in the no-capping group, while no such appearance was observed in the capping group; at 8 weeks, a typical bulbous neuroma was obtained in 10 animals (a slightly spindle shaped stump was also observed in the other 2 cases); in contrast, 100% of the capping group cases developed a bullet-shaped neuroma integrated with the conduit.

**Figure 4:**
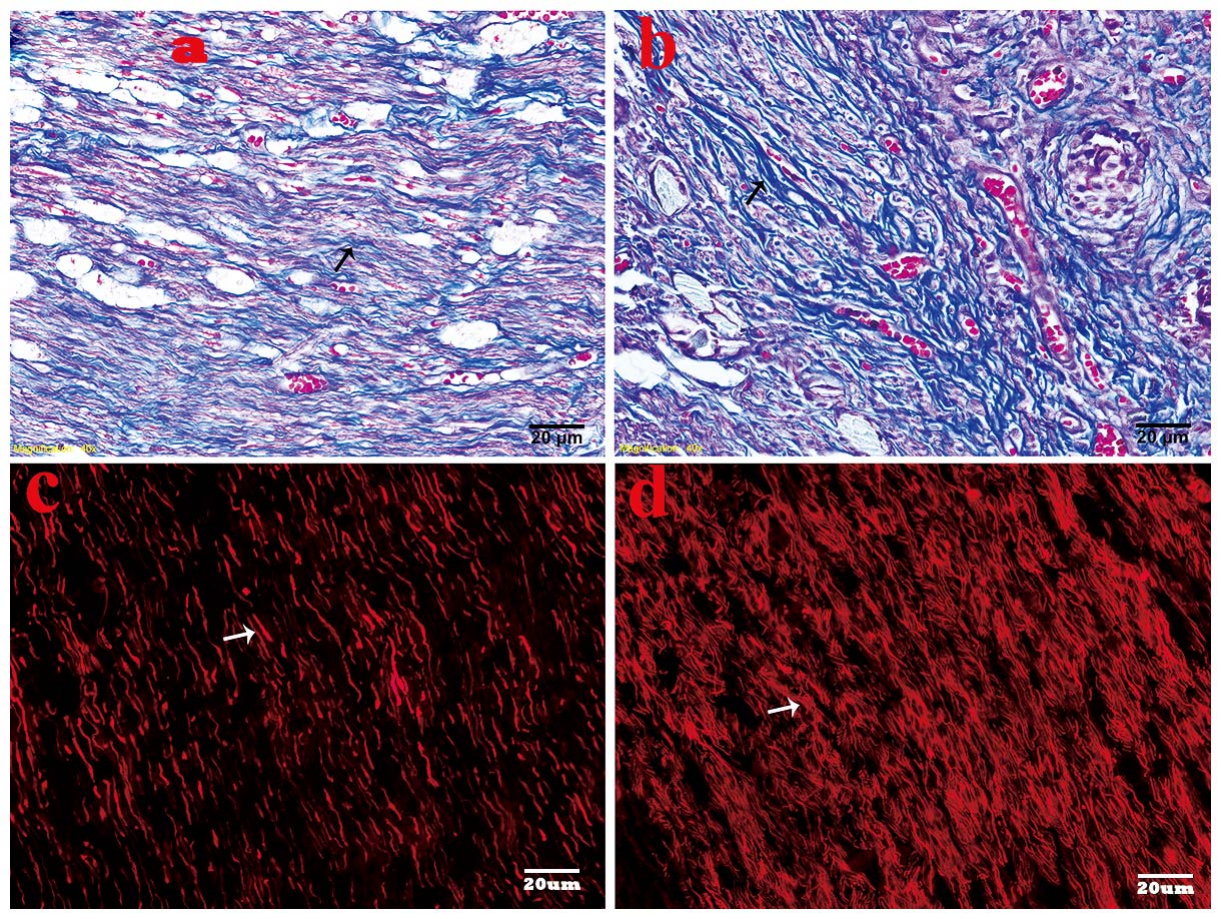
Histological analysis of neuromas at 8 weeks after surgery. A. Slightly blue stained collagens with relatively orderly arranged nerve fibers in the capping group (black arrow shows the blue collagens) by Trichrome Masson's staining. B. Dense blue stained collagens with haphazardly arranged nerve fascicles in the no-capping group (black arrow shows the blue collagens). C. The larger and medium size of axons (marked with NF-200 and stained with TRIC) arranged regularly in a linear fashion in the capping group. The arrow shows the regenerated axons. D. The regenerated axons were densely distributed in a chaotic way in the no-capping group. The arrow shows the densely-clustered axons.

Trichrome Masson's staining showed that highly proliferated collagen (stained in blue) was mingled with haphazardly arranged nerve fascicles in the no-capping group, while a relatively organized tissue feature was revealed with slightly blue stained collagens and orderly arranged nerve fibers in the capping group. (Fig. 4 a, b) In the capping group, the axons of larger (Abeta) and medium-sized fibers in the stump labeled by NF-200 using immunoflurescence method arranged regularly in a linear fashion; while in the no-capping group, the regenerated axons were densely distributed in a chaotic way. (Fig. 4 c, d) The difference in the arrangement of minifascicles was significant between the two groups (p = 0.002).

### Gene expression of MAG, MAP, PMP22, NCAM-1 and RhoA

**Figure 5:**
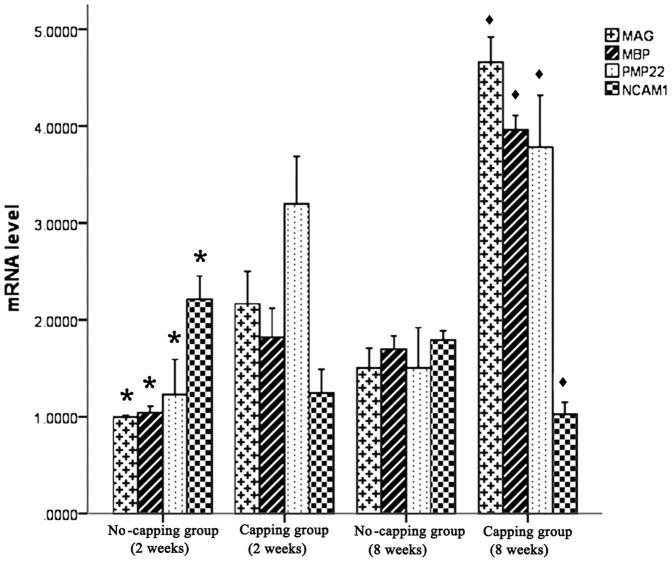
Gene expression of MAG, MBP, MP22 and NCAM-1 in neuromas. Within the two groups, real-time quantitative PCR demonstrated that the expression of myelin-specific genes: MAG, MAP and PMP22, was markedly up-regulated and the expression of NCAM-1 was down-regulated at 8 weeks compared to 2 weeks (* vs 8 weeks in the no-capping group, all p<0.001; ♦ vs 2 weeks in the capping group, all p<0.001); moreover, between the two groups, significant differences in the expression levels of the four genes were noted both at 2-week and 8-week periods (*vs capping group at 2 weeks, all p<0.001; ♦ vs no-capping group at 8 weeks, all p<0.001).

**Figure 6:**
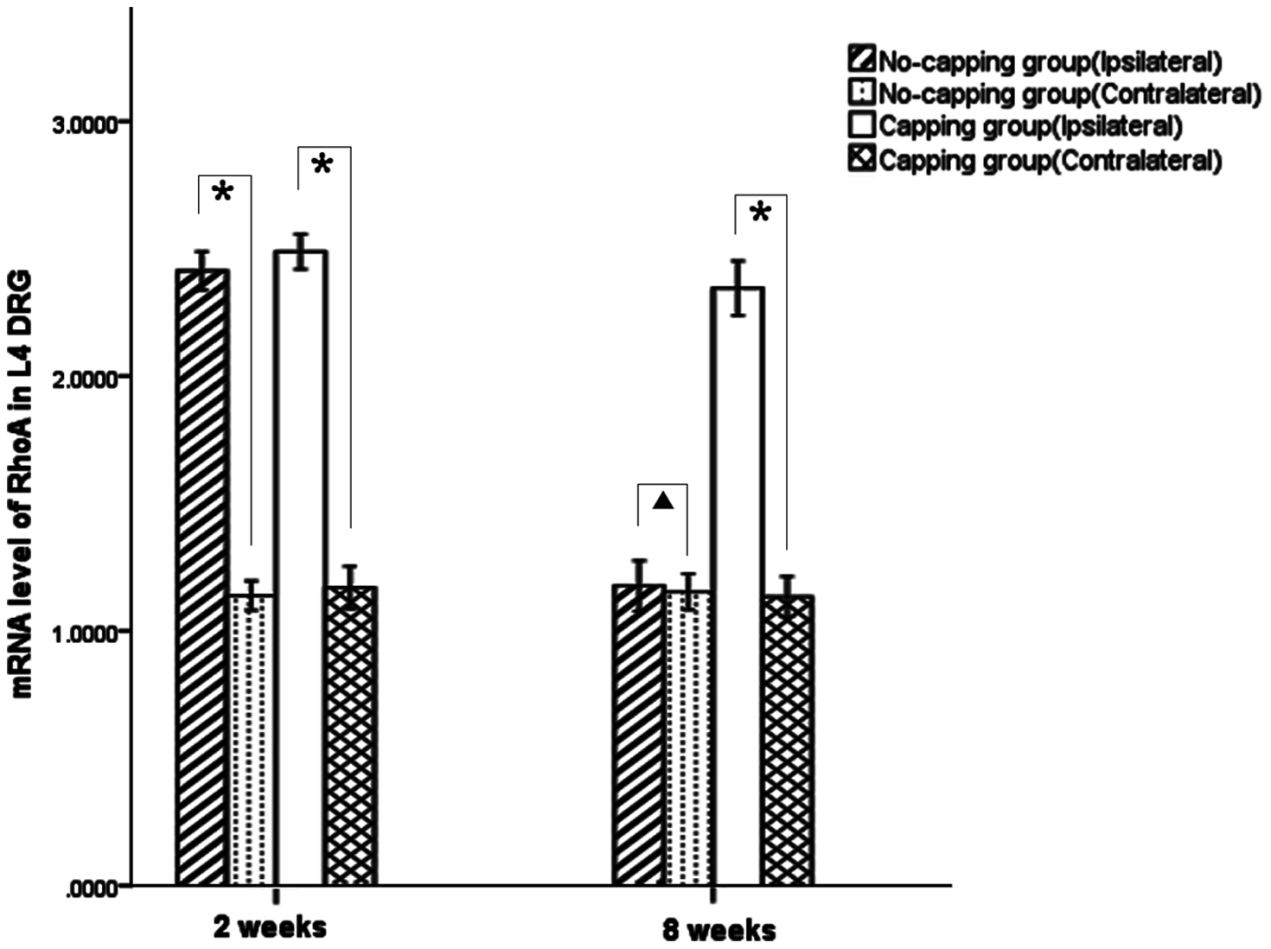
Relative mRNA expression of RhoA in the DRG (L4). The expression of RhoA in the L4 DRG was dramatically upregulated after surgery in comparison with the contralateral uninjured side at 2 weeks after surgery in both groups (both*p<0.001); however, its expression significantly dropped to the similar level of the uninjured contralateral side in the no-capping group at 8 weeks (▴p = 0.627), while it still remained in a higher level in the capping group in comparison with the contralateral side. (*p<0.001).

Real-time quantitative PCR demonstrated that the expression of myelin-specific genes: MAG, MAP and PMP22, was markedly up-regulated in the PNS 8 weeks after surgery compared to 2 weeks after surgery in both groups, while the expression of NCAM-1, which is associated with immature Schwann cells, was significantly down-regulated during this period in the two groups. Significant differences in the expression levels of the four genes were noted between the two groups both at 2 and 8 week periods. (Fig. 5) The expression of RhoA in the L4 DRG was dramatically upregulated after surgery in comparison with the contralateral uninjured side at 2 weeks after surgery in both groups (both* p<0.001); however, its expression significantly dropped to the similar level of the uninjured contralateral side in the no-capping group at 8 weeks (^▴^p = 0.627), while it still remained in a higher level in the capping group in comparison with the contralateral side. (*p<0.001). (Fig. 6)

### NGF, TGF-β1and collagen I and III detection by Western blotting assay

**Figure 7:**
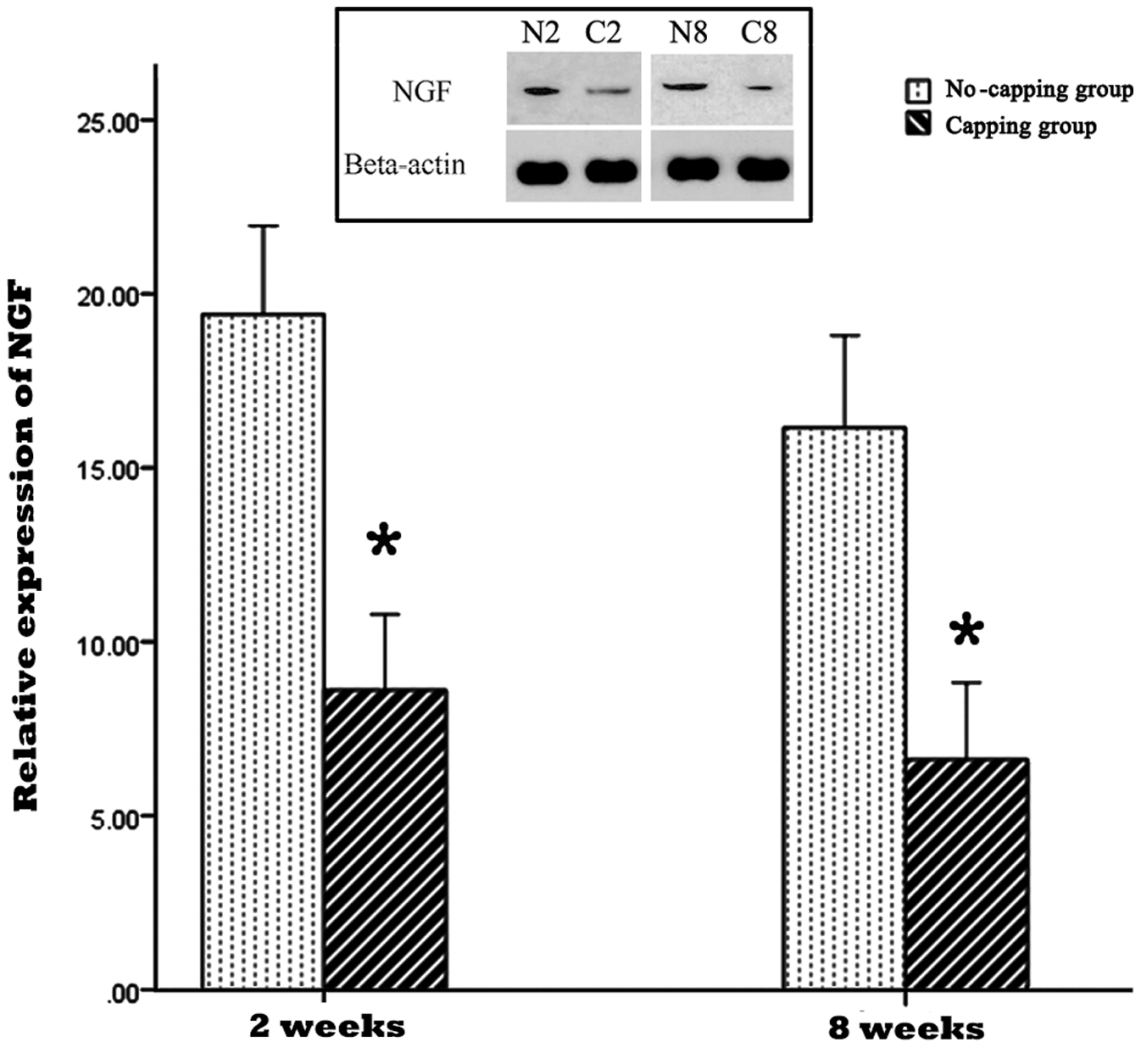
Relative expression of NGF in the neuromas. The expression of NGF was significantly lower in the capping group in comparison with the no-capping group both at 2 weeks and 8 weeks (* all p<0.001 vs no capping group). The inset picture on the top shows the immunoreactive bands from one blot, which are representative of the immunoreactive labeling in all samples. (N2: no-capping group at 2 weeks; C2: capping group at 2 weeks; N8: no-capping group at 8 weeks; C8: capping group at 8 weeks).

**Figure 8:**
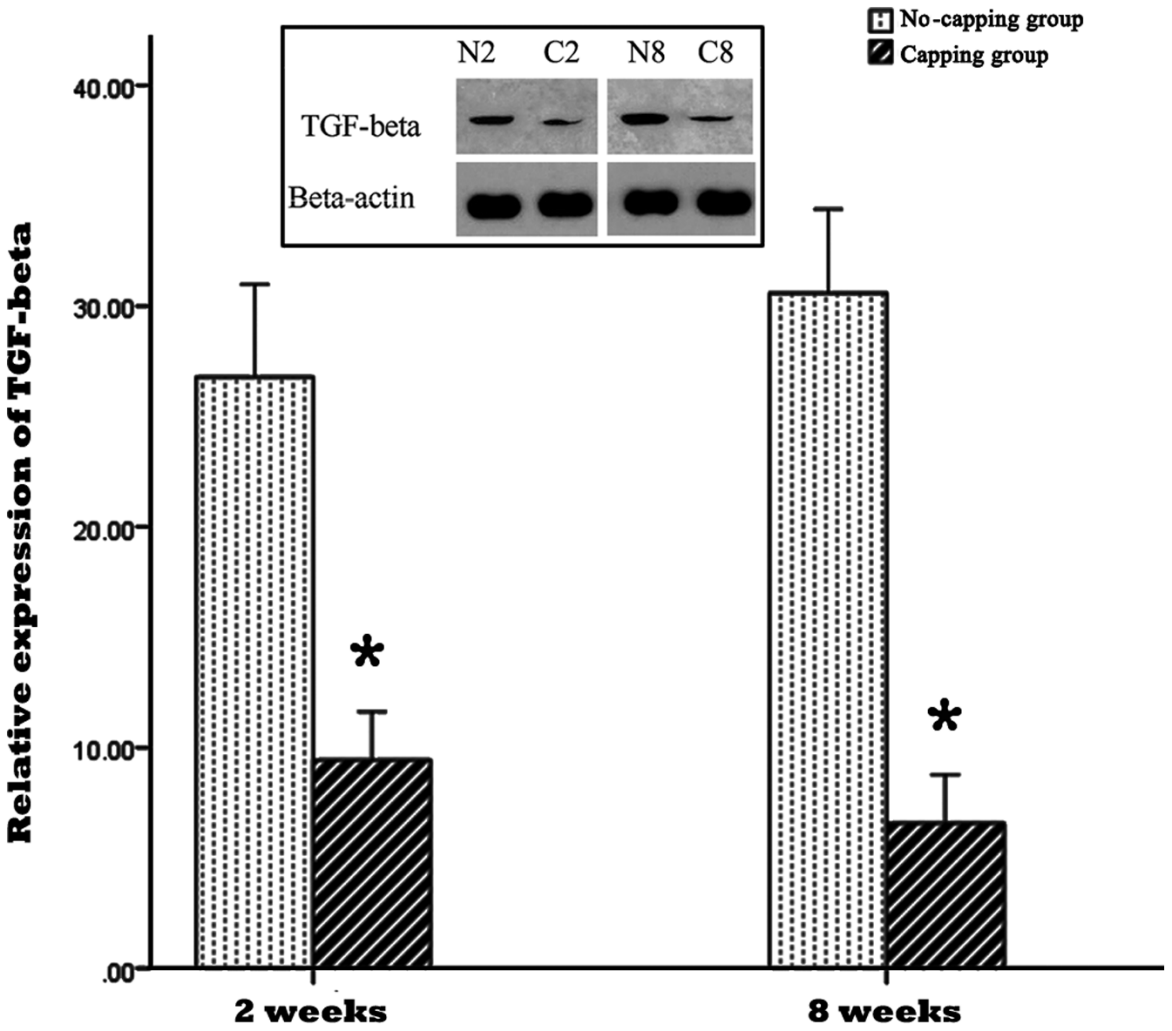
Relative expression of TGF-β1 in the neuromas. The expressions of TGF-β1 were significantly lower in the capping group in comparison with the no-capping group both at 2 weeks and 8 weeks (* all p<0.001 vs no capping group). The inset picture on the top shows the immunoreactive bands from one blot, which are representative of the immunoreactive labeling in all samples. (N2: no-capping group at 2 weeks; C2: capping group at 2 weeks; N8: no-capping group at 8 weeks; C8: capping group at 8 weeks).

**Figure 9:**
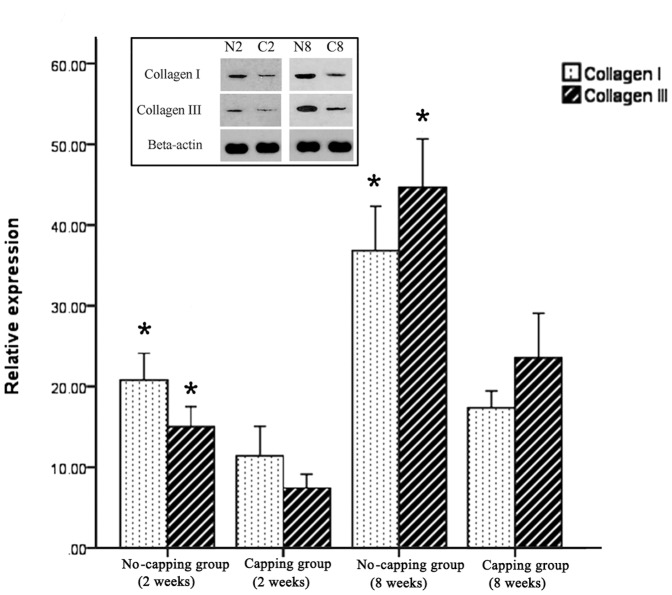
Relative expression of collagen I and collagen III in the neuromas. The protein content of collagen I and III in the no-capping group was significantly higher than that in the capping group both at 2 weeks (*p<0.001 vs capping group) and 8 weeks (♦ p<0.001 vs capping group). The inset picture on the top shows the representative immunoreactive bands from one blot. (N2: no-capping group at 2 weeks; C2: capping group at 2 weeks; N8: no-capping group at 8 weeks; C8: capping group at 8 weeks).

The result of NGF and TGF-β1 expression in the PNS by western blot was shown in Fig. 7 and 8. The expressions of NGF and TGF-β1 were significantly lower in the capping group in comparison with the no-capping group both at 2 weeks and 8 weeks. (*all p<0.001 vs no-capping group) In contrast, the protein content of collagen I and III in the no-capping group was significantly higher than that in the capping group at the two time points. (*all p<0.001 vs capping group) (Fig. 9).

### Expression of pain-related markers: c-fos and substance P

**Figure 10:**
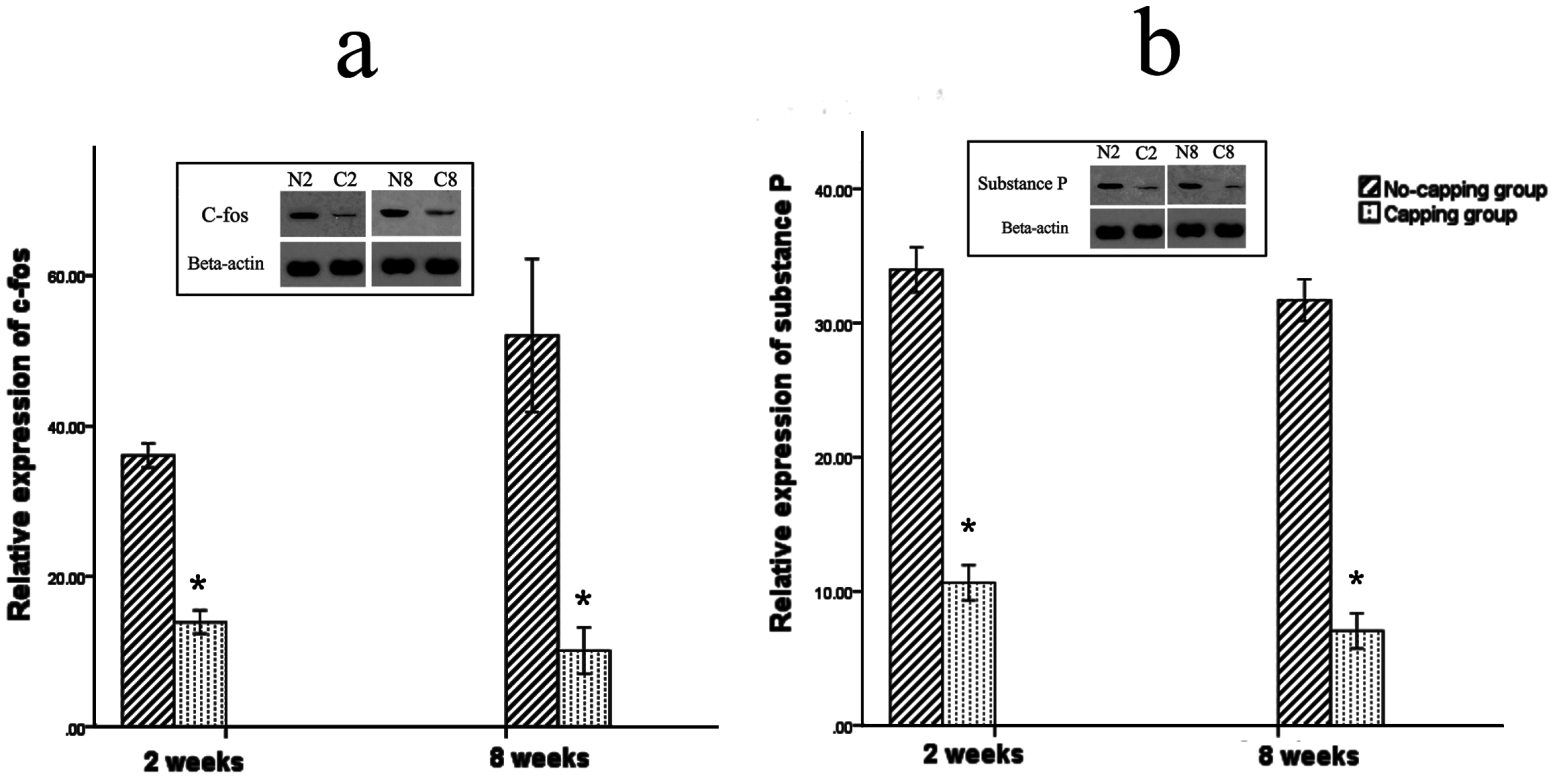
Expression of pain-related markers: c-fos and substance P. The expression of c-fos in the dorsal horn of the fourth lumbar spinal cord was significantly higher in the no-capping group than that of the capping group both at 2 weeks and 8 weeks after surgery (*both p = 0.002 vs no-capping group, Fig. 10 a). Moreover, the level of substance P in the neuromas was also higher in the no-capping group than that of the capping group either at 2 weeks or 8 weeks postoperatively (*both p = 0.002 vs no-capping group, Fig. 10 b).

The expression of c-fos in the dorsal horn of the fourth lumbar spinal cord was significantly higher in the no-capping group than that of the capping group both at 2 weeks and 8 weeks after surgery (*both p = 0.002 vs no-capping group, Fig. 10 a). Moreover, the level of substance P in the neuromas was also higher in the no-capping group than that of the capping group either at 2 weeks or 8 weeks postoperatively (*both p = 0.002 vs no-capping group, Fig. 10 b)

### Ultrastructral observation by transmission electron microscope

**Figure 11:**
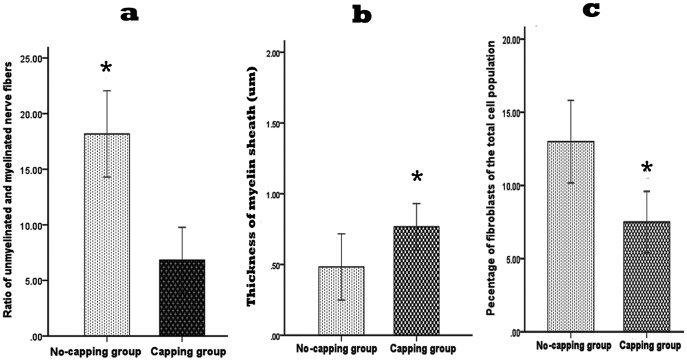
Quantitative ultrastructural analysis of the neruromas at 8 weeks after surgery. Significant higher ratio of unmyelinated and myelinated nerve fibers was observed in the neuromas of the no-capping group by TEM in comparison with that of the capping group (*p<0.001 vs capping group); the thickness of myelin sheath of the capping group was much thicker than that of the no-capping group (*p<0.001 vs no-capping group); a lower percentage of fibroblasts was seen in the capping group in comparison with that of the no-capping group (*P<0.001 vs no-capping group).

**Figure 12:**
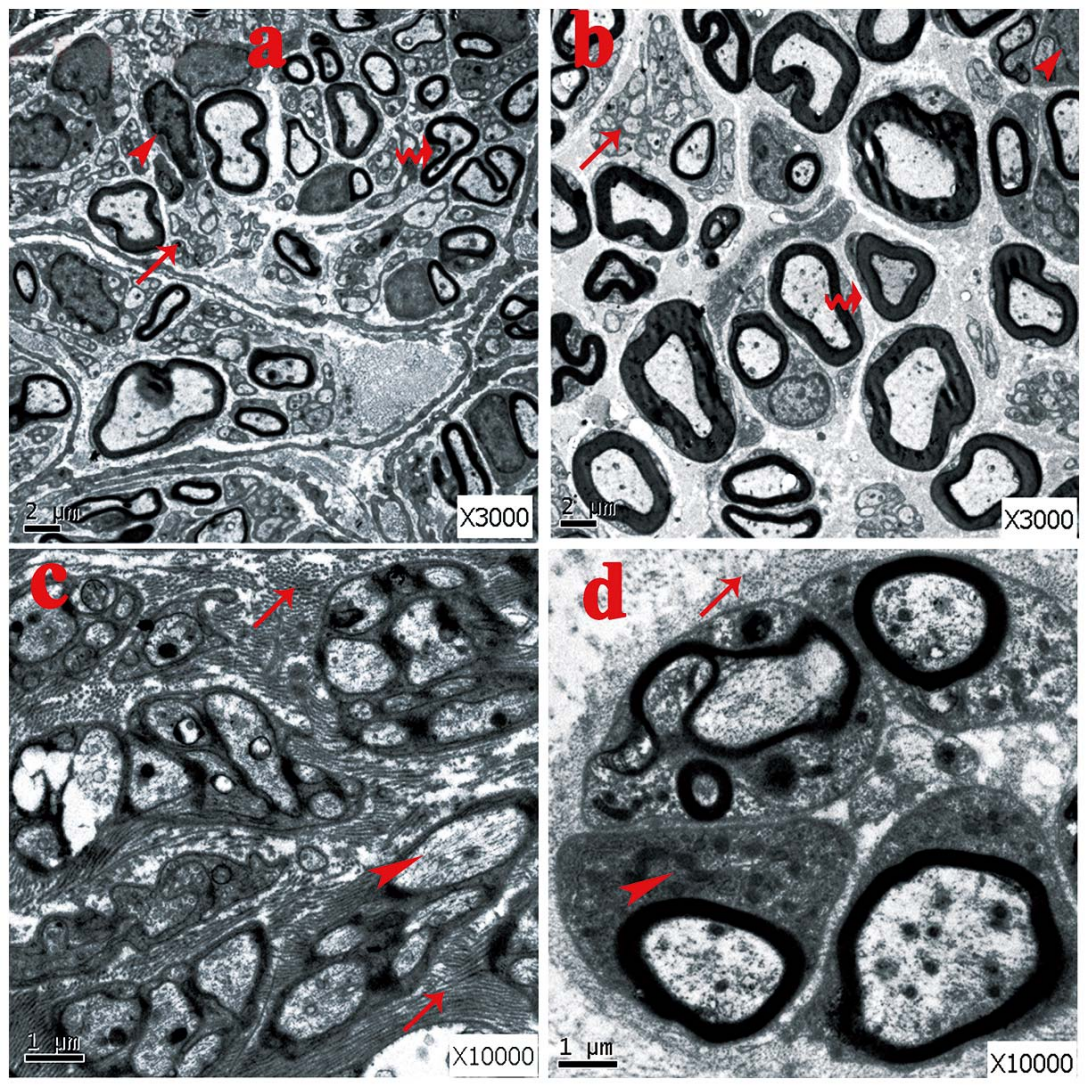
General observation by TEM at 8 weeks after surgery. A. No-capping group: many unmyelinated fibers with abundant fibroblasts were seen. The arrow head shows the fibroblast; the curved arrow shows the deformed myelinated fiber; the arrow on the bottom shows the unmyelinated fiber. B. Capping group: plenty of thick myelinated fibers with fewer fibroblasts were observed. The arrow head shows the fibroblast; the arrow in the middle shows the scattered unmyelinated fiber; the curved arrow shows the myelinated fiber. C. No-capping group: abundant transverse and oblique collagen fibers were distributed randomly. The markers show the dense transverse collagen fibers (top), Schwann cell with no recognizable organelles inside (arrow head) and oblique distributed collagen fibers (bottom). D. Capping group: simply a few transverse collagen fibers were seen around the nerve buddle. The arrow on the top shows universally distributed collagen fibers; the arrow head shows the Schwann cell with mitochondria and endoplasmic reticulum inside.

In both groups the basic structure consisted of a perineurial cell-Schwann cell-axon complex formed by groups of myelinated and unmyelinated axis cylinders surrounded by collagen fibers and fibroblasts. However, a much higher ratio of unmyelinated and myelinated fibers were seen in the no-capping group (*p<0.001 vs capping group, Fig. 11 a); the myelin sheath in the capping group were found to be significantly thicker than that in the no-capping group (*p<0.001 vas no-capping group, Fig. 11 b) In addition, fewer fibroblasts were noted in the capping group than in the no-capping group (*p<0.001 vs no-capping group, Fig. 11 c). Abundant transverse and oblique collagen fibers were distributed randomly in the no-capping group; in contrast, only a few transverse collagen fibers were seen in the capping group; furthermore, organelles such as mitochondria and endoplasmic reticulum were detectable within groups of Schwann cells in the capping group, while this was rarely found in the no-capping group (Fig. 12 a–d).

## Discussion

Nanofibrous scaffolds generated by electrospinning have gained increasing popularity in the field of tissue engineering over the past few years[28]–[30]. They are characterized by an extremely high porosity and surface area to volume ratio mimicking the features of the extracellular matrix (ECM) which is critical for tissue regeneration. Aligned nanofibers are able to provide contact guidance to cultured cells, resulting in an elongation and alignment of cells along the axes of the fibers, which plays a critical role in nerve regeneration[31]–[33]. Ceballos et al. [34] reported that aligned collagen gels provided an improved template for neurite extension compared with random collagen gels. Furthermore, Chew et al.[21] found that aligned electrospun fibers could enhance Schwann cell maturation to a greater extent than random fibers. Our previous study also showed that natural–synthetic polymeric nanofibers comprised of well-blended silk fibroin (SF) and poly (L-lactic acid-co-e-caprolactone) (P(LLA-CL)) by electrospinning could greatly improve the cell affinity of P(LLA-CL) and nerve regeneration [25]. All these findings provide fundamental basis for the present study.

The behavior of autotomy, which is triggered by neurotomy of peripheral nerve in animals and characterized as the presentation of a typical behavior of licking, scratching and self-mutilation of the denervated limb, has been regarded as an animal model of neuropathic pain-related spontaneous sensory disorders [31], [35], [36]. However, no studies have yet to convincingly confirm whether or not the observed behavior is directly related to pain [15]. Although quantitative assessment of tactile allodynia [37] or thermal hyperalgesia [38] in the rat paw is a simple method to assess the neuropathic pain in the current study, the presence of autotomy precludes this approach. Therefore, the common pain-related markers, c-fos [15], [39] and substance P [40], [41], were then selected to evaluate the pain status of the neuromas. Our findings in this study showed that the expression of c-fos in the dorsal horn of L4 spinal cord was significantly decreased in the capping group compared to the no-capping group both at 2 weeks and 8 weeks after neurectomy; on the other hand, the substance P release was also inhibited in the neuromas of the capping group when compared with that of the no-capping group. These findings imply that the capping technique may possibly prevent the neuropathic pain caused by traumatic neuromas.

It has long been known that after nerve injury, a reparative response inevitably takes place after a period of Wallerian degeneration [42]. After the period of degeneration, neurotrophic factors produced nearby (distal nerve stump, target organs, inflammatory cells, etc.) diffuse and attract regenerated nerve fibers from all directions [43], resulting in a bulb-shaped neuroma comprised of improperly and irregularly distributed nerve fibers intermingled closely with proliferated connective tissue, which additionally disperses the regenerated nerve fibers [44], [45]. This phenomenon was also noted in the proximal stump of the no-capping group in the present study. In contrast, the regenerated nerve fibers in the capping group were better-organized in comparison to the regenerated nerve fibers in the no-capping group. These results, as expected, may benefit from the contact guidance property of the conduit.

The ratio of myelinated and unmylinated fibers plays an important role in the pathological features of traumatic neuromas, which is determined by the function of the Schwann cells [16]–[18]. The general appearance of the regenerated nerve fibers in a neuroma is that of innumerable minuscule nerve fascicles with a considerably larger number of unmyelinated than myelinated fibers in painful traumatic neuromas [46]. Moreover, in a neuroma the total number of fibers is fifteen times as many as in normal nerve specimens [47]. Since pain is known to be mediated mainly by unmyelinated as well as by fine myelinated nerve fibers [48], [49], these “abnormal states” are thought to be related to the painful symptoms often seen following neuroma formation [46]. In this study, we found that there were plenty of unmyelinated fibers and a few thin myelinated fibers in the no-capping group, while in the capping group the myelinated fibers were much thicker with very few unmyelinated fibers. Based on these facts, it is conceivable that Schwann cells are “healthier” and more “mature” after capping with an aligned nanofiber tube.

In addition, the results of real-time quantitative PCR showed that the expression of MAG, MBP and PMP22, which are myelin-specific genes associated with mature Schwann cells, was significantly up-regulated; while the expression of NCAM-1, which is associated with immature Schwann cells, on the contrary, dramatically down-regulated in the capping group in comparison with the no-capping group at both 2 and 8 weeks postoperatively, indicating capping with the aligned SF/P(LLA-CL) conduit could greatly promote the maturation of the regenerated Schwann cells after neurectomy.

Following nerve injury, Schwann cells express higher levels of neurotrophic factors due to the need to establish a more conducive microenvironment for the regeneration, maintenance and regulation of neuronal function [50], [51]. Other local cells, such as fibroblasts and inflammatory cells, secret numerous other growth factors (GFs) to accelerate the wound healing process [52]. However, the western blot analysis in this study showed that the expressions of NGF and TGF-β1 in the neuroma were both significantly down-regulated at both 2 and 8 weeks after injury in the capping group as compared to the no-capping group. This result is consistent with the findings of an in-vitro study performed by Chew et al [21]: gene expression of neurotrophic factors from the seeded human Schwann cells (hSCs) on aligned fibers was down-regulated, suggesting that the hSCs have adopted a more mature phenotype on these fibers. The lower level of NGF may, therefore, inhibit the axonal elongation and perineurial cell proliferation and down-regulated TGF-β1, a key growth factor implicated in the promotion of collagen production [52], may decrease the proliferation of scar tissue. Consequently, the content of collagen I and III was much lower in the capping group than that of the no-capping group at 2 and 8 weeks after surgery and a smaller weight- ratio was also observed in the capping group 8 weeks postoperatively. The reason why there was no difference in this ratio between the two groups at 2 weeks is probably due to the early degeneration response, which was more dominant in the early stage than the regeneration progress. On the other hand, although the exact mechanism of posttraumatic pain is still unknown, many investigations support the NGF-induced theory and scar tissue contraction hypothesis [53]–[55]. Hence, our findings may partly explain the pain-relief effect of the capping technique in comparison with the no-capping group.

In this work, a high level of RhoA mRNA in the DRGs was found in both groups at two weeks after neurectomy. Surprisingly, at 8 weeks the level of RhoA mRNA in the no-capping group was decreased while it still remained significantly high in the capping group. Based on all these findings, we hypothesized that the possible mechanisms of nerve capping technique with aligned nanofiber nerve conduits might be interpreted as follows: since the transected proximal nerve stump regenerated with a relative “normal” feature (high maturation of SCs marked with high levels of MAG, MBP and PMP22 and thick myelinated fibers, etc) benefited by the capping procedure, the regeneration response to an injury was gradually eliminated and the release of cytokines, such as NGF and TGF-β1, which are usually up-regulated as a natural response to injuries during the healing process, was also inhibited as shown in the present study. Consequently, a related signal from certain peripheral cells was generated and transmitted to the peripheral neurons and a regenerative brake signal mediated by RhoA related signaling pathway (for instance, the RhoA-ROK pathway[24]) was delivered from the peripheral neurons to the peripheral effectors, resulting in the interruption of nerve regeneration. Hence, the RhoA-ROK signaling may be involved in the nerve capping procedure and might develop new strategy in the management of painful neuromas. Further research is required to verify these hypotheses.

### Conclusions

In this animal model, capping by an aligned nanofiber conduit alters the regeneration state of transected nerves and reduces painful neuroma formation, indicating a promising approach for the treatment of traumatic neuroma. The initiation of the “regenerative brake” from the peripheral neurons induced by structural as well as morphological improvements in the proximal nerve stump is hypothesized to be most likely a key mechanism for the capping technique in painful neuroma prevention.
